# Cross-language morphological transfer in similar-script bilinguals

**DOI:** 10.3758/s13423-023-02383-2

**Published:** 2023-10-26

**Authors:** Hasibe Kahraman, Bianca de Wit, Elisabeth Beyersmann

**Affiliations:** 1 School of Psychological Sciences, Macquarie University, Australian Hearing Hub, 16 University Avenue, Sydney, NSW 2109 Australia; 2 Macquarie University Centre for Reading, Macquarie University, Sydney, Australia

**Keywords:** Cross-language morphological priming, Visual word recognition, Bilingualism, Age of acquisition, Individual differences

## Abstract

The current study explored cross-language morphological transfer mechanisms using a similar-script morphological translation priming paradigm in highly proficient unbalanced Turkish (first language; L1)–English (second language; L2) bilinguals. Using noncognate English and Turkish stimuli that shared a similar meaning with no form overlap (e.g., ice [Eng.] – buz [Tur.]), in Experiment 1, L2 English stem targets (e.g., *ICE*) were primed by affixed L1 nonwords (e.g., *buzca* [*iceish*]), nonaffixed L1 nonwords (e.g., *buznak* [*iceald*]), and unrelated L1 nonwords (e.g., *tuşku [keyment]*). The results revealed priming effects in both the affixed and nonaffixed nonword conditions relative to the unrelated control, and significantly larger priming in the affixed than the nonaffixed condition. In addition, enhanced cross-language morphological transfer effects were evidenced in bilinguals with an earlier age of L2 acquisition. In Experiment 2, English stem targets (e.g., *ICE*) were primed by nonaffixed L1 nonwords including translated stems (e.g., *buznak* [*iceald*]), semantically related stems (e.g., *suzur [waterew]*), and unrelated L1 nonwords (e.g., *tuşzur [keyew]*). The results showed significantly larger priming effects in the translated condition compared with the semantic and unrelated control conditions, with no priming in the semantic condition relative to the unrelated condition, suggesting that cross-language morphological priming effects were specifically due to the lexico-semantic relationship between the embedded word and its translation equivalent.

One key theoretical milestone in visual word recognition is a widely replicated finding from masked priming, showing that briefly (50 ms) presented affixed and pseudo-affixed primes facilitate the lexical decision to their embedded stems (e.g., *farmer–FARM*; *corner–CORN*), while nonaffixed words do not (e.g., *cashew*–*CASH*), suggesting that skilled readers are experts at rapidly extracting morphological information from print (e.g., see Diependaele et al., 2005, for evidence in Dutch; Longtin et al., 2003, in French). Morphological first language (L1) priming effects have been observed across various languages (see Coughlin & Tremblay, 2015, for evidence in French; Kazanina et al., 2008 in Russian; Kırkıcı & Clahsen, 2013 in Turkish; Norman et al., 2017, in Hebrew) and stimuli types, including truly-affixed words (e.g., Beyersmann et al., 2016; Kahraman, 2022; Taft, 2004; Taft & Forster, 1976), pseudo-affixed words (e.g., Devlin et al., 2004; Longtin et al., 2003; Marslen-Wilson et al., 2008; Rastle et al., 2004), and affixed nonwords such as *farmity* (e.g., Beyersmann et al., 2013; Longtin & Meunier, 2005).

In contrast to the large body of evidence on morphological processing in L1, morphological processing in second language (L2) speakers, and specifically the mechanisms of cross-language transfer between the embedded morphemes of a bilingual’s two languages, are less well understood (for a review, see Kahraman & Beyersmann, 2023) and motivated the current investigation. While several studies have shown that bilinguals are indeed proficient at extracting morphological information from their second language, as evidenced in L2 learners of English (e.g., Feldman et al., 2010), French (e.g., Coughlin & Tremblay, 2015), Turkish (e.g., Kırkıcı & Clahsen, 2013), and Russian (e.g., Gor & Jackson, 2013), comparatively little work has investigated the mechanisms of cross-language morphological transfer between the embedded morphemic constituents of the two active languages (Kahraman & Beyersmann, 2023).

Of central importance in the investigation of cross-language morphological processing has been the masked translation priming paradigm (Chung et al., 2019; Duñabeitia et al., 2013; Kim et al., 2011; Kim & Wang, 2014; Ko & Wang, 2015; Voga, 2020; Voga & Anastassiadis-Symeonidis, 2018; Voga et al., 2020; Voga & Grainger, 2007; Wang et al., 2021; Wen & van Heuven, 2018). For example, using semantically transparent complex primes, Voga and Grainger (2007) showed that late bilinguals responded faster and more accurately to L2 French monomorphemic cognate words (e.g., *CANON* [*CANNON*]) preceded by L1 Greek affixed words (e.g., κανονιά /*kanonia*/ [*cannon*–*shot*]) relative to a phonological control condition. This initial evidence for early cross-script morphological translation priming was later replicated in Greek–French bilinguals (Voga et al., 2020) and using noncognate stimuli in Chinese–English bilinguals (Wang et al., 2021; Wen & van Heuven, 2018), Korean–English bilinguals (Chung et al., 2019; Kim & Wang, 2014; Ko & Wang, 2015).^1^ For example, Wen and van Heuven (2018) showed that lexical decisions to L2 English monomorphemic target words (e.g., *FACT*) were facilitated by prior presentation of complex Chinese translations in L1 (e.g., 事实), suggesting that the morphemic units in the two languages are activated rapidly and simultaneously in bilinguals (e.g., Duñabeitia et al., 2013; Ko & Wang, 2015; Voga & Grainger, 2007). However, at least a proportion of the previously observed priming effects can be explained by the shared semantic relationship between the prime and the target in addition to the shared morphological relationship per se (Kahraman & Beyersmann, 2023).

## The current study

To shed light on the role of orthography, morphology, and semantics in cross-language priming, the current study examined morphologically complex nonword primes constructed from illegal combinations of the L1 translations of two real embedded morphemic constituents in L2 relative to a control condition (Experiment 1), as well as the illegal combinations of L1 translations of embedded words that were semantically related to the target (Experiment 2). Since nonwords do not hold any whole-word lexical representations, the reading system relies on the identification of the embedded morphemic constituents instead. As such, the use of complex nonwords has the potential to inform distributional and decompositional theories of morphological processing. Distributional accounts view morphological structure as a by-product of orthography and semantic overlap and reframe it as a result of the statistical regularities in associations between form and meaning (e.g., Stevens & Plaut, 2022). This approach is therefore fundamentally based on the idea that morphology does not have a distinct level of representation in the mental lexicon. Decompositional accounts, on the other hand, propose morphology as an explicit level of representation where words are organized based on their morphological structure in the mental lexicon (see Amenta & Crepaldi, 2012; Marelli et al., 2020, for reviews).

Masked cross-script morphological translation priming for noncognate pairs seems robust (e.g., Schoonbaert et al., 2009; Voga & Grainger, 2007; see Wen & van Heuven, 2017, for a meta-analysis), however, it is not clear if priming for noncognate translation equivalents can be evidenced in *similar-script* languages. Only one prior study investigated masked morphological translation priming effects using noncognate words in similar-script languages (Duñabeitia et al., 2013 Spanish–English in Experiment 1 and Basque–Spanish in Experiment 2) using affixed word primes with a stimulus-onset asynchrony (SOA) of 50 ms (e.g., *doloroso* [*painful*]–*PAIN*) but found no significant priming effects. It might thus be the case that the competing orthographies of two similar-script translation equivalents (e.g., *doloroso* and *pain*) may cancel out facilitation at the level of morphology (Kim & Davis, 2003; Voga & Grainger, 2007). Since cross-language priming might require more processing depth due to the translational processes involved, the current study used 200 ms SOAs. The current study is a very first attempt at advancing our understanding of cross-language morphological processing using a carefully-matched set of complex nonword primes.

## Experiment 1

Experiment 1 sought to examine cross-language morphological processing using a masked morphological translation priming paradigm in Turkish–English^2^ bilinguals. A group of English L1 monolinguals was assessed as a control group. The goal was to clearly tease apart morphological and orthographic influences on cross-language processing using morphologically complex nonword primes. L2 English stem targets (*ICE*) were preceded by three different types of complex L1 Turkish noncognate nonword primes: affixed L1 nonwords (*buzca* [*iceish*], nonaffixed L1 nonwords (*buznak* [*iceald*]), and unrelated L1 nonwords (*tuşku [keyment]*). This experiment used morphologically complex nonword primes to prevent lexical inhibition between the prime (*iceish*) and the embedded stem (*ice*) which may have been one of the reasons for the absence of facilitation from complex real word primes (*icy*) in Duñabeitia et al.’s (2013) study. Prime–target pairs were selected such that there was no orthographic overlap. We hypothesized that if morphological information is transferred across languages, we would expect to see significant embedded stem priming effects in both the affixed and nonaffixed nonword conditions relative to the unrelated control condition. Moreover, if the presence of the affix additionally facilitates cross-language priming, we would expect to see significantly more priming in the affixed compared with the nonaffixed nonword condition. If not, we would not expect any differences between affixed and nonaffixed cross-language priming. These hypotheses were pre-registered, along with an analysis plan (https://aspredicted.org/uu5vt.pdf).

### Method

#### Participants

The required sample size for linear mixed-effects models with slightly over 80% power was determined through the use of simulation to estimate study design power within the R environment (Version 2021.09.2; R Development Core Team, 2016) using ‘simr’ package. The ‘lme4’ package was used for modelling, and the model was fitted using a competitive model testing approach and stored as the model for power analysis on inverse response times (RTs). Based on this analysis, 72 advanced L2 speakers of English (50 females, age: *M* = 30.72 years, *SD* = 4.79, range: 21–43) and 70 L1 speakers of English (57 females, age: *M =* 22.8 years, *SD* = 7.35, range: 17–47) participated in the experiment in exchange for monetary reimbursement or course credit. Bilinguals were all late learners of English with a high level of English proficiency (see Table 1 for participant demographics).

**Table 1:** Participant demographics

Variable		*M*	Range	max	*M*	Range	max
		L1 GROUP (*N* = 70)			L2 GROUP (*N* = 72)		
Years^a^	of education	12.85	1–19		18.41	5–24	
	Spent in an Eng.-speaking country	22.7	14–47		3.25	0–23	
Age of	First contact with Eng.^b^	1.31	1–3		10.18	3–22	
	First reading Eng.^c^	4.16	1–7		12.35	6–25	
	Fluent reading Eng.^d^	6.66	3–16		16.32	7–27	
Level of proficiency in^e^	Speaking Eng.	9.88	8–10	10	8.37	5–10	10
	Understanding Spoken Eng.	9.85	8–10	10	8.8	5–10	10
	Reading in Eng.	9.78	7–10	10	8.98	5–10	10
Current exposure to Eng. in^f^	Interacting with friends	10	10	10	7.12	0–10	10
	Interacting with family	9.81	6–10	10	2.73	0–10	10
	Watching TV	9.75	6–10	10	7.97	0–10	10
	Listening to radio/music	9.78	6–10	10	7.89	0–10	10
	Reading	9.98	9–10	10	8.16	3–10	10
	Language/Lab instruction	9.79	0–10	10	5.5	0–10	10
Eng.	AoA^g^	4.47	1–15		18.16	9–27	
	Accentedness^h^	0.65	0–10	10	5.46	0–10	10
	Nonnativeness^i^	0.57	0–10	10	6.41	0–10	10

*Eng* English, *AoA* age of acquisition

^a^Number of years of formal education and number of years spent in an English-speaking country

^b^Age when participants began acquiring English

^c^Age when participants began reading in English

^d^Age when participants became fluent in reading English

^e^Participants rated their proficiency on a rating scale from 0–10 on the domains of speaking, understanding spoken English, reading in English, where 0 and 10 denoted to “none” and “perfect,” respectively

^f^Participants rated to which extent they were exposed to English on a rating scale from 0–10 in interacting with friends and family, watching TV, and listening to radio/music, reading, and language-lab/self-instruction, where 0 and 10 denoted to “none” and “always,” respectively

^g^Age at which participants became fluent in English. We believe that this definition is more realistic given that participants were not immersed in the foreign language at the time of the testing

^h^Based on how much of a foreign accent a participant has in English in their own perception

^i^Based on how frequently others identify participants as a nonnative speaker based on their accent in English

Five L1 English participants who reported having language or learning disabilities or having acquired English at a late age, and 10 L2 participants who were identified as nonnative speakers of Turkish or who acquired English early in life were excluded prior to the analyses. Participant demographics were collected via The Language Experience and Proficiency Questionnaire (LEAP-Q; Marian et al., 2007). Only some components of LEAP-Q were used since this shorter version was more feasible in an online data collection setting where participants were more likely to be affected by environmental distractors with lengthy tasks. This short questionnaire asked participants to list all languages they know in order of dominance and in order of acquisition. They were also asked to list the percentage of the time they were exposed to each language and to name the cultures with which they identify. The number of years participants spent in an English-speaking family and a school and/or working environment was additionally asked. The rest of questions as well as participant responses are presented in Table 1.^3^

#### Materials

For the lexical decision task, 90 L2 English stem targets (e.g., *ICE*) were selected as targets. The list of word trials is reported in Appendix 1 (see also https://osf.io/snxm5/ for a full set of materials). It was ensured words were frequent (*M* = 4.67, *SD* = 0.58), concrete noncognate items with no pseudo-suffixes. Word frequencies were extracted from the SUBTLEX-UK database (van Heuven et al., 2014) using the Zipf values. Concreteness ratings were obtained from Brysbaert et al. (2014) (*M* = 4.54, *SD* = 04.69, range: 3.16–5). Of 90 targets, 70 were nouns, 11 verbs, and nine adjectives. English target words were translated into Turkish. The translated Turkish translations acted as stems embedded in affixed and nonaffixed prime stimuli. The English words and their Turkish translations were matched on number of phonemes (*M* = 3.3 for English targets; *M* = 3.5 for Turkish translations, *p* = .26) and, though less robustly, on length (*M* = 4 for English targets; *M* = 3.7 for Turkish translations, *p* = .08). However, the number of syllables of English words was lower than their Turkish translations (*M* = 1.04 for English targets; *M* = 1.37 for Turkish translations, *p* < .0001), something that is caused by structural differences between the Turkish and English languages (e.g., Turkish words are easier to break into syllables; Durgunoğlu & Öney, 1999).

Affixes and nonaffixes (–*CA*^4^ vs. –*KI*) were matched on number of letters and positional specific bigram frequency (P23). It was ensured that nonaffixes would not stand as a stem and would always be part of a real word (e.g., *cash*–*ew*). Like nearly all other suffixes^5^ in Turkish, six suffixes used in current study had more than one form on basis of the vowels that precede it, which exhibits one form of vowel harmony (see Table 2 for suffix details). In addition to these vowel alternations, consonants in suffixes are also subject to alternations on basis of voicing assimilation rule, which requires the devoicing of an initial voiced consonant of a suffix (i.e., c, d, g turns into ç, t, k, respectively) when the base ends in a voiceless consonant (f, s, ş, h, p, ç, t, k). Therefore, six different suffixes were repeated 15 times in different forms (e.g., –CA; –ca, –ce, –ça, –çe^6^) in the affixed nonword condition and 5 times in each list.

**Table 2:** Turkish suffixes, their English translation equivalents, and their meanings

Turkish	Alternations	English	Examples	Meaning
–lIk	lık/lik/luk/lük	–ness	iyi–iyilik/good–goodness	Added to nouns or adjectives to make abstract nouns.
–sIz	sız/siz/suz/süz	–less	ümit–ümitsiz/hope–hopeless	Added to nouns to form adjectives.
–CA	ca/ce/ça/çe	–ish	çocuk–çocukça; Türk– Türkçe / child–childish; Turk–Turkish	Added to nouns to create adjectives, describing actions or attitudes. It also creates nouns, adjectives or adverbs denoting a language from nouns of nationality.
–CI	cı/ci/cu/cü/ çı/çi/çu/çü	–er	iş––işçi; içki––içkici / work––worker; drink––drinker	Added to nouns to form nouns indicating a person associated with a profession or indicating a person engaged in a particular activity.
–lI	lı/li/lu/lü/	–ful	dikkat––dikkatli/ care––careful	Added to nouns or adjectives to make nouns or adjectives which denote (1) possessing the object or quality indicated by the basic word, (2) possessing the object or quality in a high degree, (3) belonging to a place or institution.
–GI	gı/gi/gu/gü/ kı/ki/ku/kü	–ion, –ment^a^	bil––bilgi; sor––sorgu / know––knowledge; inquire–– inquisition	Added to verbs to form nouns.

^a^There is no direct equivalent of –GI suffix in English. Therefore, these are some translation options

Once the stems were combined with potential endings, the affixed and nonaffixed Turkish nonwords were matched on positional specific bigram frequency (suffixed nonwords: *M* = 4,709, *SD* = 2,933; nonsuffixed nonwords: *M* = 4,022, *SD* = 1,966).

Stems in the unrelated condition (e.g., *tuş*) were selected such that they closely matched the stem in the related condition (e.g., *buz*) on length, word frequency, bigram frequency, number of phonemes, number of syllables, orthographic neighbourhood, and OLD20. Half of the unrelated stems were combined with affixes, the other half with nonaffixes, thus mirroring the experimental design used for related nonwords.

For the purposes of the lexical decision task, 90 nonword filler stimuli were created by changing one to two letters in real English words (e.g., *end*–*enp*). Real word and nonword targets were matched on length and bigram token frequencies. They were not matched on the orthographic neighbourhood size or positional specific bigram frequencies, however, due to the need to control for the other experimental factors. Their Turkish primes were constructed by combining a new set of Turkish nonwords that were constructed by changing one to two letters in real Turkish words (e.g., *yut* [*swallow*]–*kuf*) with the same affixes and nonaffixes as for the experimental words. Three experimental lists were created using a Latin square design, each list containing only one of the prime-target pairs to ensure each participant responded to each target only once.

English L2 target words were preceded by three different types of complex L1 Turkish nonword primes (see Table 3 for characteristics): affixed L1 nonwords (e.g., *buzca* [*iceish*], nonaffixed L1 nonwords (e.g., *buznak* [*iceald*]), and unrelated L1 nonwords (e.g., *tuşku [keyment]*).

**Table 3:** Mean item characteristics (*SD*s)

	English targets	Their Turkish translations
No .of letters	4 (0.7)	3.7 (0.99)
No. of phonemes	3.3 (0.69)	3.5 (0.89)
No. of syllables	1.04 (0.20)	1.37 (0.48)
	Related stem (e.g., *buz [ice*])	Unrelated stem (e.g., *tuş [key])*
Length	3.68 (0.99)	3.70 (1)
Word freq.^a^	388.36 (1.279)	527.23 (2.366)
Bigram freq.	2,760 (1,902)	2,962 (2,018)
No. of phonemes	3.46 (0.89)	3.43 (0.93)
No. of syllables	1.37 (0.48)	1.48 (0.52)
Orth. N.	11 (7.58)	12.24 (8.50)
OLD20	1.40 (0.36)	1.36 (0.38)
Concreteness	4.54 (0.46)	4.50 (0.46)
	Affixes (e.g., –*CA*)	Nonaffixes (e.g., –*NAK*)
No. of letters	2.2 (0.4)	2.2 (0.4)
P23	1,095 (1,224)	967.13 (1,152)

^a^Frequencies for these translated stems used as primes were extracted from BOUN Corpus of 490 million words (Sak et al., 2011)

#### Procedure

Participants were tested online using the Gorilla platform (https://gorilla.sc). Each trial consisted of a 500-ms fixation cross, 500-ms forward mask of hash keys, followed by a 200-ms prime in lowercase, then the uppercase target. The target remained on screen until the response was made or until 3 seconds elapsed. Participants were instructed to respond as quickly and accurately as possible. After the lexical decision task, participants also responded to a short version of LEAP-Q (Marian et al., 2007) to ensure that participants are late bilinguals who speak Turkish as their L1 and English as their L2, and that they are proficient in their L2.

#### Analysis

All data and analyses script are available (https://osf.io/snxm5/?view_only=b3115a5d31f0431d89ce955a526407e5). Response time (RT) analysis was conducted on correct trials only. As a criterion for removal of extreme outliers, RT distributions as well as error rates for items and subjects were visually inspected separately for L1 English and L2 English groups. Three participants from each group were removed since their nonword response times or accuracies were two standard deviations above the mean. Individual data points below 250 ms or above 2,000 ms were also removed (L1: 0.4 %; L2: 0.5 % of the total data). Data points whose standardized residuals were greater than 2.5 standard deviations (Baayen, 2008) were removed (L1: 2.8 %; L2: 3.2 %). Table 4 shows mean RTs and error rates (ERs) across conditions for the L1 and L2 speakers of English. The results focused on response times only because the ERs were very low (see Table 4). The significance level is set at 0.05.

**Table 4:** Mean response times (RTs) and error rates (ERs) across conditions, in ms (*SD*s)

	Affixed nonword	Nonaffixed nonword	Unrelated nonword
Example	*buzca–ICE*	*buznak–ICE*	*tuşku–ICE*
L2 English group			
RT	667 (138)	677 (145)	696 (159)
Priming effect	29*	19*	
ER	0.01 (0.13)	0.03 (0.17)	0.03 (0.18)
L1 English group			
RT	643 (121)	643 (123)	645 (127)
Priming effect	2	2	
ER	0.02 (0.16)	0.02 (0.15)	0.02 (0.15)

The asterisk (*) indicates a significant post-hoc test at *p* < .05

The L1 and L2 datasets were then merged, and a Box-Cox power transformation indicated RTs be inverse-transformed for the merged analysis of data to normalize RT distributions and reduce the effect of outliers. Priming effects were tested in a mixed-effect model which included the two fixed effects of group (L1, L2) and of prime type (affixed nonword, nonaffixed nonword, unrelated) using ANOVA Type III (Version 3.0-12; Fox & Weisberg, 2019). Trial number was normalized and added as an additional predictor to the model to discard the effects of fatigue or habituation. The data were modelled in a model with reduced random slope structure^7^ (Barr et al., 2013).

### Results and discussion

The main effects of group and prime type were significant, *F*(1, 137.95) = 7.32, *p* < .001; *F*(2, 136.41) = 8.14, *p* < .0001, respectively, with a robust two-way interaction between group and prime type, *F*(2, 132.14) = 10.81, *p* < .0001, suggesting that priming effects differed across L1 and L2 groups. To further unpack the interaction between prime type and group, we analyzed the effect of prime type separately for each participant group.

Statistical analyses were conducted on inverse-transformed RTs for the L2 group, while no transformation was required for the L1 group on basis of Box-Cox power transformations. Linear mixed-effect modelling was used to fit RTs and response accuracies for both groups. The model for each group contained prime type (affixed nonword, nonaffixed nonword, unrelated) as a fixed effect and subjects and items as random effects factors (random intercepts). Trial number was normalized and also added as an additional predictor to the model to discard the effects of item presentation. The data was modelled in a maximal random slope structure (Barr et al., 2013), and pairwise comparisons between conditions were computed using the emmeans package (Lenth, 2019).

The main effect of prime type was non-significant in L1 English speakers, χ^2^(2) = 0.80, *p* = .66. However, the model for L2 English speakers yielded a robust main effect of prime type, χ^2^(2) = 36.91, *p* < .0001. Trial order was a significant covariate in L1 speakers, χ^2^(1) = 4.15, *p* = .041, and L2 speakers, χ^2^(1) = 37.37,* p* < .0001. The pairwise comparisons of factor prime type for L2 speakers are presented in Table 5. Lexical decisions to words preceded by affixed and nonaffixed related nonword primes (e.g., *buzca*–*ICE*; *buznak*–*ICE*) were significantly faster than in the unrelated control condition (*z* = −6.06, *p* < .0001; *z* = −3.77, *p* < .001), demonstrating cross-language embedded stem priming effects. Facilitation of embedded stems occurred independently of the morphemic structure (i.e., for both affixed and nonaffixed nonwords) due to the lexical activation of the translated stem (i.e., *buzca [iceish]–ice*) in both conditions. In addition, there was a significant difference between the affixed and nonaffixed nonword prime conditions (*z* = −2.47, *p* = .01), showing that the presence of the affix additionally facilitated cross-language priming. This result suggests that cross-language morphological priming occurred in absence of whole-word access and orthographic overlap.^8^

**Table 5:** Pairwise contrasts between prime types in the L2 group

Contrast	Estimate	*SE*	*df*	*z* ratio	*p* value
Affixed–unrelated	−0.0567	0.00935	Inf	−6.063	<.0001
Nonaffixed–unrelated	−0.0374	0.00991	Inf	−3.770	<.001
Affixed–nonaffixed	−0.0193	0.00783	Inf	−2.470	.01

Inf = Infinity

#### AoA and L2 priming

**Fig. 1:**
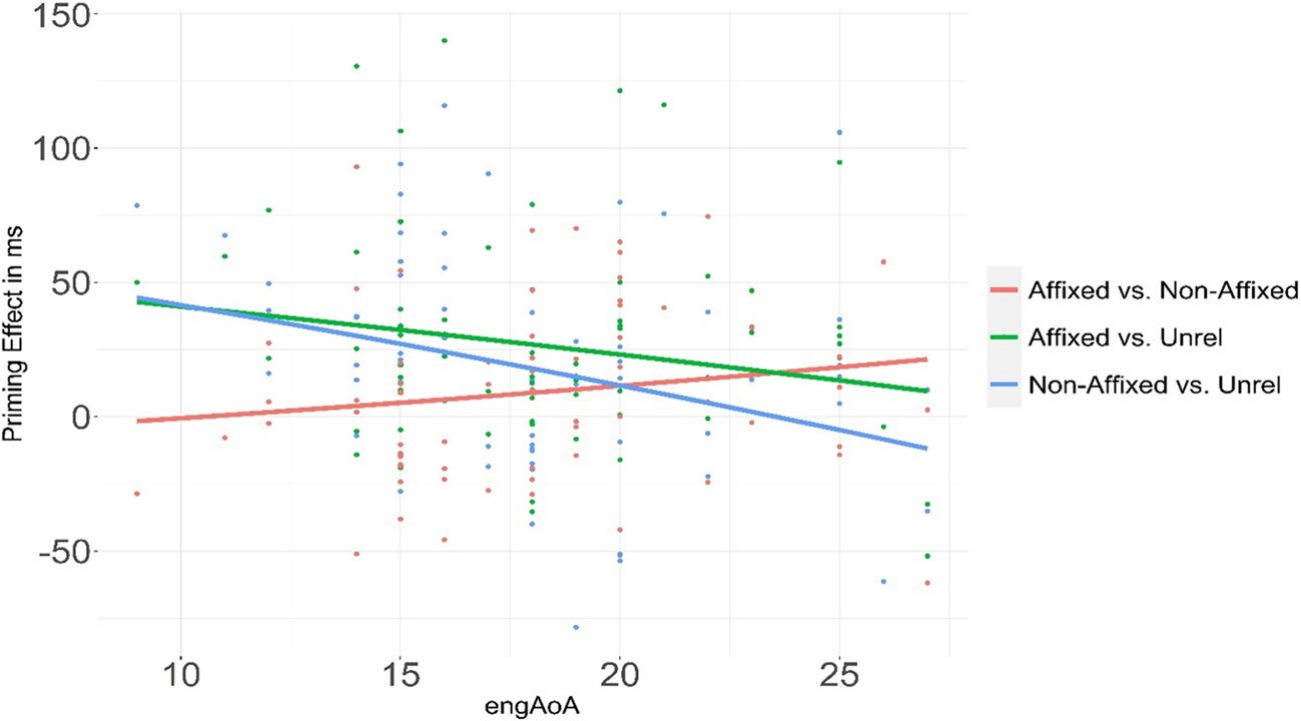
Priming effects (in milliseconds) as a function of age of acquisition (EngAoA in years). *Note*. All three priming effects interacted significantly with AoA. Green and blue lines indicate affixed priming (e.g., *buzca [iceish]*) and nonaffix priming (e.g., *buznak [iceald]*), whereas the red line shows participants’ sensitivity to morphological structure in the stimuli. (Color figure online)

A more exploratory, non-preregistered analysis of the L2 data was performed in order to assess the modulation of cross-language morphological priming by age of English acquisition (EngAoA), which previous studies have shown is an important predictor for cross-language priming (e.g., Sabourin et al., 2014). Even though all bilinguals of this study were characterized as late bilinguals (see Table 1 for participant characteristics), we could still examine the effect of AoA by comparing bilinguals who acquired the language at an earlier age versus later age. This variable was added to the model^9^ to calculate the interaction with factor prime type. The interaction between prime type and EngAoA was computed using the emmeans package. Results demonstrated a two-way interaction, *χ*^*2*^(2) = 11.10, *p* = .003, showing that prime type was modulated by age of L2 acquisition. Further post hoc analysis comparing earlier and later acquirers of English in the form of 0.05 and 0.95 quantiles (i.e., 12 and 25 years of age; Table 6) revealed that the interaction was due to more robust priming effects in participants who acquired English at an earlier age (Fig. 1). Critically, the difference between the affixed and nonaffixed conditions increased with increasing EngAoA (the red line, Fig. 1), suggesting that late acquirers were more reliant on morphological structure.

**Table 6:** Priming as a function of age of acquisition (EngAoA) comparing earlier (E) and later (L) acquirers of English with estimates for each contrast, standard errors (*SE*), degrees of freedom (*df*), z ratio, and *p* values

Contrast	Estimate	*SE*	*df*	*z* ratio	*p* value^a^
Unrelated vs. affixed EvL	0.1096	0.0183	Inf	5.955	<.0001
Unrelated vs. nonaffixed EvL	0.0683	0.0190	Inf	3.589	<.001
Affixed vs. nonaffixed EvL	0.0413	0.0157	Inf	2.624	<.01

^a^All *p* values are adjusted using the Holm method for multiple comparisons

Inf = Infinity

In conclusion, the results of Experiment 1 provide evidence for significant morphological translation priming with both L1 affixed and nonaffixed nonword primes towards the visual recognition of L2 stem targets. Affixed nonword primes yielded significantly more facilitation than nonaffixed primes. However, the size of the affixed and nonaffixed priming varied as a function of the age of L2 acquisition. Late acquirers of L2 English were more reliant on morphological structure, while early acquirers were primarily guided by the identification of embedded words, a point we return to in the General Discussion.

## Experiment 2

The purpose of this experiment was to investigate the extent to which cross-language embedded word translation priming effects are affected by semantics at 200 ms SOA. L2 English stem targets (e.g., *ICE*) were preceded by three different types of L1 Turkish primes. Critically, the stimuli were composed of nonaffixed stimuli to simulate a priming manipulation that is less biased towards morphemic parsing.^10^ In the translation condition, targets were preceded by L1 nonwords with embedded words that were noncognate translation equivalents of the target (e.g., *buznak [iceald]*). Naturally, the embedded stem and its translation equivalent also shared a semantic relationship. In the semantic condition, targets were preceded by L1 nonwords with embedded words that were semantically related to the target (e.g., *suzur [waterew]*), but did not represent direct translations. In the control condition, targets were preceded by L1 unrelated nonwords (e.g., *tuşzur [keyew]*)*.* Note that the nonaffixed L1 translated condition was identical to Experiment 1. If cross-language priming is purely due to the shared semantic relationship between the embedded word of the prime and the target, we would expect significantly greater priming in the semantic relative to the unrelated condition, and greater priming in the translated than the semantic condition. If, however, cross-language priming is specifically due to the lexico-semantic relationship that is shared between the Turkish stems and their English target translation equivalents, we would still predict greater priming in the translated compared with the semantic condition, but crucially, priming in the semantic versus unrelated condition should be absent.

### Method

#### Participants

Seventy-six advanced Turkish–English bilinguals (34 females, age: *M* = 30.76 years, *SD* = 4.95, range: 19–46) participated in the experiment in exchange for monetary reimbursement or course credit. Using participant responses to LEAP-Q, seven participants who reported having acquired Turkish at a late age and had a low proficiency in Turkish, or who acquired English early in life were excluded prior to the analyses. Participant responses are presented in Table 7.

**Table 7:** Participant demographics

Variable		*M*	Range	max
		(*N* = 69)		
Years	of education	19.28	4–26	
	Spent in an Eng.-speaking country	3.27	0–13	
Age of	First contact with Eng.	10.03	4–18	
	First reading Eng.	12.49	7–28	
	Fluent reading Eng.	16.54	8–30	
Level of proficiency in	Speaking Eng.	8.29	5–10	10
	Understanding Spoken Eng.	8.86	6–10	10
	Reading in Eng.	9.16	7–10	10
Current exposure to Eng. in	Interacting with friends	7.05	2–10	10
	Interacting with family	2.14	0–10	10
	Watching TV	7.52	2–10	10
	Listening to radio/music	7.21	0–10	10
	Reading	8	3–10	10
	Language/lab instruction	5.44	0–10	10
Eng.	Accentedness	5.80	1–10	10
	Nonnativeness	6.43	0–10	10
	AoA	18.06	11–30	

#### Materials

L2 English targets (e.g., *ICE*) were preceded by nonaffixed L1 translated nonwords (e.g., *buznak [iceald]*), nonaffixed L1 semantically related nonwords (e.g., *suzur [waterew]*), and nonaffixed L1 unrelated nonwords (e.g., *tuşzur [keyew]*). The translated nonword condition was identical to Experiment 1. The semantic primes were selected by retrieving (and then translating) semantic associations of the English target words from the Small World of Words (smallworldofwords.org; De Deyne et al., 2019)*.* All items were concrete words (nouns, verbs, or adjectives) that contained no pseudo-suffixes. These were then combined with the same nonaffixes as in the translated nonword condition. The Turkish translations and semantic associations were matched on length (*M* = 3.07; *M* = 3.08, *p* = 0.3, respectively), frequency^11^ (*M* = 392.6; *M* = 200.3, *p* = .2, respectively), number of syllables (*M* = 1.4; *M* = 1.5, *p* = .1, respectively), number of phonemes (*M* = 3.5; *M* = 3.6, *p* = 0.4, respectively), OLD20 (*M* = 1.4 *M* = 1.4, *p* = 0.3, respectively), orthographic neighbourhood (*M* = 11.1; *M* = 10.5, *p* = .6, respectively), and positional specific bigram frequency (*M* = 90.9; *M* = 98.3, *p* = .7, respectively). The full list of stems is reported in Appendix 2.

Once the stems were combined with 30 potential endings, nonword stimuli in the three conditions were matched on length (*M* = 5.9, *M* = 6.0,* M* = 5.9, respectively). For the purposes of the lexical decision task, 90 nonword filler stimuli from Experiment 1 were combined with the same nonaffixes as the experimental words. Three experimental lists were created using a Latin square design, each list containing only one of the prime–target pairs to ensure each participant responded to each target only once.

#### Procedure

The procedure and analysis were the same as in Experiment 1. The planned analyses were preregistered prior to data collection (https://aspredicted.org/sc5ky.pdf). A Box-Cox power transformation of the RTs indicated RTs not be transformed (please refer to the following OSF link for all data and analyses script: https://osf.io/sy4d8/?view_only=66a20e7007c946b88147f9d7f16f3416).

### Results and discussion

The main effect of prime type was significant, χ^2^(2) = 13.70, *p* < .001. Trial order was also a significant covariate, χ^2^(1) = 7.70, *p* < .001. Table 8 shows mean RTs and error rates across conditions. Similar to Experiment 1, the results in Experiment 2 focused on RTs only since error rates (ERs) were very low.

**Table 8:** Mean response times (RTs) and error rates (ERs) across conditions, in ms (*SD*s)

	Nonaffixed translated nonword	Nonaffixed semantically related nonwords	Nonaffixed unrelated nonword
Example	*buznak–ICE*	*suzur–ICE*	*tuşzur–ICE*
RT	646 (145)	656 (151)	664 (150)
Priming effect	18*	8	
ER	0.02 (0.16)	0.03 (0.16)	0.03 (0.17)

The asterisk (*) indicates a significant post-hoc test at *p* < .05

The pairwise comparisons of factor prime type for L2 speakers are presented in Table 9. Lexical decisions to words preceded by nonaffixed translated nonword primes (e.g., *buznak–ICE*) were significantly faster than in the unrelated control condition (*z* = −3.69, *p* < .001), demonstrating cross-language embedded priming effects. In addition, there was a significant difference between the nonaffixed translated and nonaffixed semantically related nonword conditions (*z* = 2.46, *p* = .02). The difference between the semantic and the unrelated conditions was not significant (*z* = −1.15, *p* = .24), demonstrating that semantic prime–target overlap alone was not sufficient to trigger priming.

**Table 9:** Pairwise contrasts between prime types in Experiment 2

Contrast	Estimate	*SE*	*df*	*z* ratio	*p* value^a^
Translation–unrelated	−17.05	4.62	Inf	−3.695	<.001
Semantic–unrelated	−4.86	4.2	Inf	−1.158	.24
Semantic–translation	12.19	4.95	Inf	2.463	.02

^a^All *p* values are adjusted using the holm method for multiple comparisons

Inf = Infinity

Although priming in the semantic condition did not differ statistically from the unrelated condition, it is possible that priming in the translated condition might have been at least partially due to the shared semantic relationship between the stem of the prime (*buz*) and the target (*ice*), which was proportionately larger in the translated than in the semantic control condition. However, as we discuss in more detail below, what really seems to be driving morphological translation priming effects is the direct lexico-semantic relationship between the embedded word and its translation equivalent. This second experiment thus sheds further light on the nature of cross-language morphological priming by providing important insights into the shared lexical and semantic contributions in this process.

## General discussion

Experimental evidence reported in the current study showcased for the first time the potential of morphologically complex nonwords and nonmorphological embedded words as effective noncognate translation primes in similar-script bilinguals. The results of Experiments 1 and 2 showed that robust noncognate cross-language embedded word and morphological priming effects were present in similar-script bilinguals. We focused our cross-language investigation on morphologically complex nonword stimuli, including affixed and nonaffixed nonwords in Experiment 1 as well as nonaffixed semantically related nonwords in Experiment 2, to disentangle the separate effects of stems and affixes.

### Morphological influences on cross-language processing

Several key conclusions can be derived from the current findings. First, priming effects observed in Experiment 1 in the affixed (*buzca–ICE*) and nonaffixed (*buznak–ICE*) nonword conditions relative to the unrelated condition (*tuşku–ICE*) suggest that bilinguals were able to rapidly extract the stems embedded in the Turkish L1 nonword primes and map them onto their corresponding L2 (English) translations, which in turn facilitated their L2 lexical decision response. Although primes (which were all nonwords) in this study were visible (200 ms SOA), it is clear that the identification of affixes in this task was due to a prelexical embedded stem activation given that the nonword primes did not correspond to existing whole-word lexical representations.

Second, cross-language activation was further facilitated by the activation of the L1 Turkish affix in Experiment 1, as evidenced by the significantly larger priming effects in the affixed compared with the nonaffixed condition. The here reported cross-language morphological priming effects show that bilinguals are sensitive to morphological structure of words and that they are experts at rapidly extracting the stem (*buz*) embedded in a nonword prime, independently of whether it was accompanied by a real affix (*buzca*) or a nonaffix (*buznak*), which then in turn leads to the activation of its stem’s translation equivalent (*ice*), thus producing target word facilitation. Third, given that none of the prime–target pairs shared any orthographic relationship in either experiment, it can be ruled out that the observed priming effects were simply due to lower-level form overlap between the two languages.

Fourth, the results of Experiment 2 revealed significant priming in the translated (*buznak [iceald]–ICE*) but not in the semantic condition (*suzur [waterew]–ICE*) relative to the unrelated condition, with a statistically significant difference between the translated and semantic conditions. These findings indicate that semantics alone is not sufficient to trigger cross-language morphological effects (for similar arguments, see Voga, 2020), and instead suggest that bilinguals were able to map the stems embedded in the L1 primes onto the lexical representations of the translated L2 stem targets. It is possible that semantic relatedness partially contributed to cross-language embedded word priming effects in the translation condition at 200 ms SOA, as has been previously shown to be the case in cross-language priming studies with monomorphemic words (e.g., Schoonbaert et al., 2009).

Duñabeitia et al.’s (2013) study was the first to examine cross-language priming in Spanish–English (similar-script) bilinguals; they, however, failed to report significant priming effects in their noncognate stimuli using 50 ms SOA. The absence of facilitation in their study may perhaps be explained by some level of competition between the lexical representations of the Spanish primes (*doloroso*) and the English target words (*pain*), thus counteracting any potential facilitation that may have come from the presence of the affix (–*oso*). In the two experiments, these potentially competing mechanisms were avoided since none of the primes formed existing real words. Also, Duñabeitia and colleagues used a relatively short prime duration, which may have been too brief to trigger cross-language morphological priming effects. In the current study, via exposing primes for a longer duration, participants were allowed more time to thoroughly process the prime thereby increasing the magnitude of morphological facilitation (Feldman, 2000).

### Cross-language priming as a function of age of L2 acquisition

The results further showed that cross-language morphological co-activation is more pronounced in bilinguals with earlier age of L2 acquisition. One possible explanation for the AoA influences on L2 priming is that morphological organization of lexical representations differs in different bilingual groups. Bilinguals who acquired English at earlier ages could effectively map sublexical orthography in L1 Turkish onto full-form orthographic representations in L2 English, and therefore show less sensitivity to the morphological information in the L1 stimuli, resulting in similar magnitudes of priming from affixed and nonaffixed nonwords. Bilinguals with less experience, on the other hand, employ visual word recognition processes that are guided more by the processing of L1 affixes, potentially because these individuals may be applying morphological parsing strategies from their dominant agglutinative Turkish L1. This finding adds to the growing number of studies showing that individual variability in AoA determines the strength of lexical connections between morphemic constituents in the bilingual lexicon (e.g., Dimitropoulou et al., 2011; Sabourin et al., 2014).

### Theoretical implications, limitations, and conclusion

**Fig. 2:**
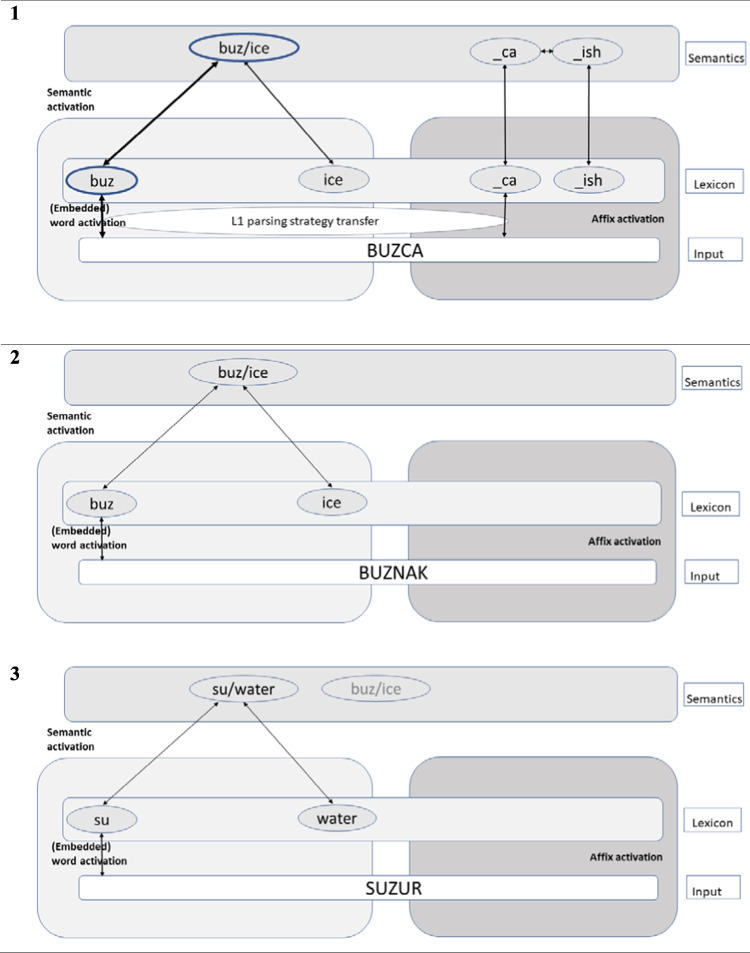
Hypothetical flow of activation in bilingual complex word identification system for affixed nonwords (**Panel 1**), nonaffixed nonwords (**Panel 2**), and nonaffixed semantically related nonwords (**Panel 3**). *Note*. Activation between layers flows via bidirectional connections. Stronger connections are indicated via thicker lines, and stronger activations are indicated via thicker rings. This mimics Turkish–English bilinguals who speak similar-script languages. The visual recognition of complex words in bilinguals occurs in a three-layered network of orthographic input, orthographic lexicon, and semantic representations. In visual input ‘*buzca*’ in the top panel (Engl. ‘*iceish*’), its morphemic constituents (*buz, –ca*) as well as translation equivalents (*ice, –ish*) are activated simultaneously within an integrated orthographic lexicon. The nonaffixed nonword ‘*buznak’* in the middle panel (Engl. ‘*iceald*’) simultaneously activates the embedded word (*buz*) as well as its translation equivalent (*ice*) within an integrated orthographic lexicon. The additional facilitation for affixed (*buzca*) compared with nonaffixed nonwords (*buznak*) is explained by Turkish–English bilingual’s sensitivity to morphological structure in the processing of complex nonwords in L1 Turkish (indicated via “L1 parsing strategy transfer” from their agglutinative native language to English in Panel 1). Whenever a letter string is exhaustively decomposable into morphemes (e.g., *buz + ca*), the reading system rises the activation levels of the corresponding morphemes, which explains the comparatively larger priming effects in the affixed compared with the nonaffixed nonword conditions. As shown in Panel 3, embedded words (e.g., *su* in *suzur* [Engl. ‘*water’* in ‘*waterew’*]) will strongly activate their corresponding semantic representations that is shared between languages (‘su’/’water’) which in turn will be mapped onto the lexical representation of the translation equivalents in L2. In addition, the embedded word (*su*) will weakly activate other semantic neighbours (e.g., the semantic representation of ‘buz’/’ice’; indicated by a node in opaque grey colour). The latter point provides a hypothetical explanation for the absence of ‘*suzur*–*ICE*’ priming in our data

The current findings do not allow us to clearly tease apart the assumptions of distributional theories of morphological processing (e.g., Amenta et al., 2020; Plaut & Gonnerman, 2000; Stevens & Plaut, 2022) whereby semantics and morphology are inevitably intertwined, and decompositional theories of morphological processing (e.g., Beyersmann & Grainger, 2023; Crepaldi et al., 2010; Grainger & Beyersmann, 2017; Taft, 2004) that view morphemes as being independently represented in the reading system. However, given the study’s focus on cross-language morphological transfer in bilinguals, we will centre the below discussion primarily around existing interactive activation models of bilingual word recognition. The observed cross-language priming effects from affixed and nonaffixed nonword primes are broadly consistent with current bilingual models of lexical access such as the Bilingual Interactive Activation + (BIA+ model; Dijkstra & van Heuven, 2002) and Multilink models (Dijkstra et al., 2019) that propose an integrated mental lexicon enabling the rapid simultaneous activation of bilinguals’ two languages. However, although the BIA+ and Multilink models provide a sophisticated approach to addressing cross-language mechanisms of monomorphemic word identification, based on the parallel, simultaneous activation of the two languages within a single integrated lexicon, they are less able to capture the processing of morphologically complex words. Given the underspecification of existing cross-language processing theories with regard to the identification of morphologically complex words, Kahraman and Beyersmann (2023) proposed a tentative theoretical framework integrating the basic principles of the word and affix model (Beyersmann & Grainger, 2023; Grainger & Beyersmann, 2017) and Multilink model (see Fig. 2).

Within this bilingual network, it is proposed that there are two distinct mechanisms running in parallel towards the processing of a complex word. While stem activation is handled by the non-morphological embedded word activation mechanism (e.g., *buz* in *buzca* and *buznak*), the morphological affix activation mechanism triggers the activation of affixes (e.g., –*ca*; see top panel in Fig. 2). Importantly, both mechanisms operate bottom-up simply by mapping letter strings onto existing lexical and affix representations in the orthographic lexicon. The activation of stems tolerates minor orthographic alterations. A given stimulus input activates words with slight orthographic deviations in addition to those that provide an exact match with the input. Importantly, the proposed mechanisms operate within a single integrated bilingual lexicon where the Turkish morphemes (*buz, –ca*,) as well as their English translation equivalents (*ice, –ish*) are simultaneously activated, thereby facilitating the recognition of target words as a real word in the other language than the prime. Activated units that share relevant meaning representations in both languages then send and receive activation from the semantic level. Critically, activation flows between layers via bidirectional excitatory connections. As can be seen in Fig. 2, the here observed cross-language embedded word priming effects are captured in the bidirectional links between the semantic representation and the lexical representation of the translated stem. Given that relationships between translation equivalents in this model are mediated via semantics, it is further predicted that the magnitude of cross-language morphological priming depends on the degree of semantic overlap between L1 prime and the L2 target at 200 ms SOA (see Panel 3 in Fig. 2 for the processing of nonaffixed semantically close nonwords that failed to activate the target *ICE*^12^).

An important question for future research is the question of automaticity of cross-language morphological transfer effects. The exploration of cross-language priming effects with shorter SOAs or the simultaneous recording of event-related brain potentials (ERPs) and behavioural measures within a cross-language morphological priming study may be able to shed light on the time-course of cross-language activation processes in bilinguals. Another prospect for future work lies in the investigation of cross-language transfer effects using translation equivalent affixes, rather than translation equivalent morphemic stems. Although the current data clearly provide evidence for cross-language priming of stem targets, it is less clear if these results generalize to cross-language transfer between the affix representations of L1 and L2, which would provide further evidence for a highly specialized morphemic transfer system that bilinguals can draw on in their reading. In addition, a replication of our Experiment 2 with semantically related words embedded in affixed nonword primes (e.g., *suca* [*waterish*]–ICE) using a longer prime duration might yield further insights into the independent contribution of lexical, morphological, and semantic influences on cross-language morphological translation priming effects. Finally, it remains to be seen if the here observed priming effects generalize to other languages with a less productive morphology than Turkish. It has been reported that morphological processing in L1 changes as a function of morphological productivity of a language (e.g., Beyersmann et al., 2020, 2021; Mousikou et al., 2020). Examining the influence of language-specific differences in morphological complexity therefore represents an important avenue for the investigation of cross-language morphological transfer effects in bilinguals.

In sum, the two experiments reported in the current study provided evidence for the dynamic interplay between morphological representations across languages in similar-script bilinguals. A general trend towards more robust cross-language morphological priming was observed in early compared with late bilinguals, thus pointing to the important role of individual variability in bilingual research (e.g., Andrews & Lo, 2012; Kahraman & Kırkıcı, 2021). The observed difference between affixed and nonaffixed nonword priming demonstrates that bilinguals with a morphologically complex L1 employ a word recognition strategy that is sensitive to morphological information in L1 Turkish and highlights the need for further specifications of current bilingual models of visual word recognition to integrate morphological information. We here propose a tentative theoretical bilingual framework to accommodate cross-language morphological transfer effects. Further work is required to test the generalizability of the proposed theoretical constraints to other languages.

## Data Availability

The analysis scripts for Experiments 1 and 2 are available (https://osf.io/snxm5/?view_only=b3115a5d31f0431d89ce955a526407e5) and (https://osf.io/sy4d8/?view_only=66a20e7007c946b88147f9d7f16f3416), respectively.

## Appendix 1

### English word targets and corresponding Turkish nonword primes in Experiment 1

**Table:**

TARGET	Affixed nonword	Nonaffixed nonword	Unrelated word
ICE	buzca	buznak	tuşku
ARM	kolca	kolzu	çizlik
SAND	kumca	kumba	solku
SOAP	sabunca	sabunza	çorbaşa
OIL	yağca	yağıf	dizfi
WIG	perukça	perukzu	çocukpa
SKIN	ciltce	ciltep	biraca
HAND	elce	ellev	atlık
NIGHT	gecece	gecefi	kedigi
MINT	nanece	nanehi	kafaza
JAM	reçelce	reçelpe	kılıçzı
SWEAT	terce	terih	güznel
BUG	böcekçe	böcekşe	arabagı
MEAT	etçe	etçer	illik
DOG	köpekçe	köpekpe	barajkı
CHIN	çeneci	çenefe	sahacı
BALD	kelci	kelbi	sürlü
TEXT	metinci	metinşi	kafesfe
DAMP	nemci	nempe	topvu
PINK	pembeci	pembehi	mısırıf
POET	şairci	şairzi	köşelev
FOX	tilkici	tilkişi	göğüspe
SNOW	karcı	kardız	boksuz
NEST	yuvacı	yuvafi	mavipe
FOG	sisçi	sisnel	kanpa
LAKE	gölcü	gölçer	kasça
DAY	güncü	günep	vursuz
ASH	külcü	külnel	birci
FACE	yüzcü	yüzep	dalba
FLY	uççu	uçza	işpi
FIG	incirgi	incirbi	dosyaca
KING	kralgi	kralıf	gripçi
TENT	çadırgı	çadırzı	buharca
TEA	çaygı	çaydız	çitki
SALT	tuzgu	tuzşa	bezçer
ROAD	yolgu	yolvu	çiğfi
ROSE	gülgü	güllev	hapkı
COAT	ceketki	ceketşi	kadroku
ROPE	ipki	ipzi	evgi
WINE	şarapkı	şarapzı	tabakıç
CLASS	sınıfkı	sınıfpa	sahnezi
CROWN	taçkı	taçıf	bağva
STONE	taşkı	taşpa	çamlev
TRASH	çöpkü	çöpnel	şallık
MILK	sütkü	sützü	tıknak
YOUNG	gençli	gençfi	gagalık
GO	gitli	gitih	silce
CUT	kesli	keshi	devlik
DUCK	ördekli	ördekih	pipetpi
ROUGH	sertli	sertpi	fareşe
AUNT	teyzeli	teyzeşe	silahpi
HORN	kornalı	kornaba	pamukça
GRAVE	mezarlı	mezarpi	meyveşi
BURN	yaklı	yakıç	kızdız
HEART	kalplik	kalpıç	kareci
SPOT	lekelik	lekeşe	kölebi
FLOOD	sellik	selbi	telze
EAT	yelik	yefe	ayba
BELL	zillik	zilpi	çekze
GLASS	camlık	camba	köklük
LIP	dudaklık	dudakpu	nehirli
WALL	duvarlık	duvarza	armutça
DOT	noktalık	noktapa	parçaba
HAIR	saçlık	saçıç	kışzı
PIG	domuzlu	domuzvu	vitesçi
STOP	durlu	durzur	dulsuz
COLD	soğuklu	soğukvu	köprüzü
BLIND	körlü	körçer	çölcü
KISS	öplü	öpzü	içnel
WALK	yürülü	yürüze	cücehi
SWAN	kuğuluk	kuğupu	cadıcı
SKY	göklük	gökze	çimki
BURY	gömlük	gömfe	gözce
FUR	kürklük	kürkze	annegi
WOOL	yünlük	yünlev	çişfe
WEB	ağsız	ağdız	izki
KNIFE	bıçaksız	bıçakva	çorapça
CROP	kırpsız	kırpzı	babakı
SHELF	rafsız	rafşa	kilçer
PAGE	sayfasız	sayfaşa	nesnefe
HOT	sıcaksız	sıcakva	teknepe
BACK	sırtsız	sırtva	aynaza
BLACK	siyahsız	siyahzi	külahşi
YEAR	yılsız	yılpa	çiypi
EMPTY	boşsuz	boşzur	baslık
RUN	koşsuz	koşpu	üstkü
WOLF	kurtsuz	kurtzur	popoku
BIRD	kuşsuz	kuşnak	yazca
VOTE	oysuz	oyzu	unzur
DUST	tozsuz	toznak	beşpe

## Appendix 2

### English word targets and corresponding Turkish stem primes in Experiment 2

**Table:**

Stem target	Semantic stem [English translation]
ICE	su [water]
ARM	bacak [leg]
SAND	iri [coarse]
SOAP	pak [clean]
OIL	dök [spill]
WIG	vekil [barrister]
SKIN	ben [mole]
HAND	sağ [right]
NIGHT	akşam [evening]
MINT	ot [herb]
JAM	dut [berry]
SWEAT	ıslak [moist]
BUG	vız [hum]
MEAT	kasap [butcher]
DOG	tazı [hound]
CHIN	gamze [dimple]
BALD	rahip [monk]
TEXT	söz [word]
DAMP	küf [mold]
PINK	renk [colour]
POET	mısra [verse]
FOX	av [hunt]
SNOW	dağ [mountain]
NEST	yurt [home]
FOG	pus [mist]
LAKE	havza [dock]
DAY	sabah [morning]
ASH	is [soot]
FACE	çil [freckle]
FLY	ez [swat]
FIG	hurma [date]
KING	taht [throne]
TENT	çivi [peg]
TEA	kahve [coffee]
SALT	deniz [sea]
ROAD	cadde [street]
ROSE	çiçek [flower]
COAT	kat [layer]
ROPE	lif [string]
WINE	kadeh [goblet]
CLASS	ders [course]
CROWN	bey [prince]
STONE	mıcır [flint]
TRASH	zırva [garbage]
MILK	keçi [goat]
YOUNG	tay [foal]
GO	ak [flow]
CUT	doğra [chop]
DUCK	banyo [bath]
ROUGH	düz [smooth]
AUNT	hısım [relative]
HORN	düdük [honk]
GRAVE	türbe [tomb]
BURN	parla [flame]
HEART	saat [ticker]
SPOT	kir [blot]
FLOOD	tufan [deluge]
EAT	gıda [food]
BELL	çan [ding]
GLASS	ampul [lightbulb]
LIP	kıl [(facial) hair]
WALL	sıva [plaster]
DOT	harç [dab]
HAIR	kaş [eyebrow]
PIG	in [sty]
STOP	fren [brake]
COLD	don [freeze]
BLIND	engel [handicap]
KISS	kucak [embrace]
WALK	yaya [pedestrian]
SWAN	kaz [goose]
SKY	dış [outside]
BURY	deş [dig]
FUR	tüy [fuzz]
WOOL	triko [knitting]
WEB	örgüt [network]
KNIFE	aygıt [utensil]
CROP	biç [harvest]
SHELF	depo [storage]
PAGE	kitap [book]
HOT	ılık [warm]
BACK	ön [front]
BLACK	kömür [coal]
YEAR	doğ [born]
EMPTY	tam [full]
RUN	kaç [escape]
WOLF	ulu [howl]
BIRD	kumru [dove]
VOTE	ses [voice]
DUST	hav [lint]

## Footnotes

1 Greek–French, Korean–English, and Chinese–English language pairs use different scripts.

2 Turkish and English are similar-script languages.

3 Before testing, all participants signed a written consent.

4 Affixes that are subject to alternations are indicated in capital letters.

5 The terms “suffix” and “affix” are used interchangeably in the present study.

6 The abundance of grammatical cases, indicated by different affixes of varying length and vowel harmony shows that Turkish is a morphologically rich language.

7 The lmer syntax is: -1000/RT ~ prime.type2*group + scale(TrialN) + (prime.type2+ scale(TrialN) |ID) + (prime.type2+group+ scale(TrialN)|Trial.Item), ALLPPT, control=lmerControl(optimizer="bobyqa", optCtrl=list(maxfun=2e5),calc.derivs=F)

8 Some of our nonword primes included stem–suffix concatenations that may have been semantically interpretable in Experiment 1. To test the potential influence of semantic interpretability, we conducted a more exploratory, non-preregistered analysis by which all nonword primes were screened and classified as interpretable or noninterpretable, and then added as a covariate to the reported linear mixed-effects models. The results confirmed that the inclusion of semantic interpretability did not change the direction or significance of the reported findings (for a detailed summary of the results, see https://osf.io/snxm5/?view_only=b3115a5d31f0431d89ce955a526407e5).

9 The effect of EngAoA in LEAP-Q was assessed in a separate model. The lmer syntax is as follows: -1000/RT ~ prime.type2 * EngAoA+ scale(TrialN) + (prime.type2 + scale(TrialN)|ID) + (prime.type2 + scale(TrialN)|Trial.Item),w1,control=lmerControl(optimizer="bobyqa",optCtrl=list(maxfun=2e5),calc.derivs=F)

10 We thank the editor for this suggestion.

11 Frequencies were extracted from BOUN Corpus of 490 million words (Sak et al., 2011).

12 Schoonbaert et al. (2009) obtained significant cross-language semantic priming effects in monomorphemic words using 250 ms SOA, but not 100 ms SOA. Hence, the recognition of semantically related words might require more processing depths, which might be achieved by presenting primes for a longer duration such as 250 ms.
